# Cicatricial Organizing Pneumonia with Dendriform Pulmonary Ossification: An Unusual Cause for a Recurrent Pneumothorax

**DOI:** 10.1155/2019/2379145

**Published:** 2019-12-09

**Authors:** Mnahi Bin Saeedan, Carol Farver, Atul C. Mehta, Ruchi Yadav

**Affiliations:** ^1^Section of Thoracic Imaging, Imaging Institute, Cleveland Clinic, Cleveland, OH, USA; ^2^Department of Pathology, University of Michigan, Ann Arbor, MI, USA; ^3^Department of Pulmonary and Critical Care Medicine, Cleveland Clinic, Cleveland, OH, USA

## Abstract

Cicatricial organizing pneumonia is an uncommon form of organizing pneumonia, which may manifest as persisting linear opacities on computerized tomography (CT) scan mimicking a fibrosing interstitial pneumonia. It may also manifest with pulmonary ossification, which is a metaplastic bone formation within the lung tissue. The latter presentation could be either nodular or dendriform, both secondary to underlying lung disease and rarely idiopathic. Dendriform pulmonary ossification (DPO) has rarely been described as a cause of spontaneous pneumothorax. We present a case of a 55-year-old male with history of recurrent pneumothoraces and worsening dyspnea on exertion. A CT of the chest revealed progressive bilateral sub-pleural and peribronchovascular reticular opacities associated with densely ossified branching and nodular opacities. Video-assisted thoracoscopic biopsy of the lung demonstrated cicatricial organizing pneumonia with areas of marked diffuse DPO. The case highlights that dendriform pulmonary ossification arising from cicatricial organizing pneumonia should be considered in the differential diagnosis of recurrent pneumonias among patients with lower lobe sub-pleural reticular opacities. The case highlights that dendriform pulmonary ossification rarely can cause spontaneous pneumothorax and can be associated with cicatricial organizing pneumonia and reticular opacities on imaging.

## 1. Introduction

Organizing pneumonia (OP) is characterized by the presence of organizing fibromyxoid proliferations within the lumens of respiratory bronchioles and alveolar ducts. Peripheral and /or peribronchiolar consolidations are the most frequent findings of OP on a computerized tomography (CT) scan. These opacities could be migratory in nature. OP is often steroid-responsive and reversible with total resolution of radiological opacities, but occasionally may recur [1, 2].

Cicatricial OP (OPc) is a newly described entity in the pathology literature [3, 4] and distinguished from conventional OP by formation of irreversible dense fibrous bands and small nodules in the background of conventional OP [4]. The reported CT findings of OPc are variable and range from typical imaging findings of OP to lower lobes predominant peribronchovascular and subpleural reticular opacities with or without pulmonary ossification [4]. The latter is characterized by metaplastic ossification in the lung and is classified into nodular (NPO) and dendriform (DPO) subtypes [5]. Dendriform pulmonary ossification is usually described in association with chronic lung disease including usual interstitial pneumonia (UIP) [5, 6] although it can be seen in isolation [7, 8]. Dendriform pulmonary ossification presenting with spontaneous pneumothorax has been reported in a few occasions [9–14] but rarely with OPc [4]. We report a case of cicatricial organizing pneumonia with DPO who presented with recurrent pneumothorax. The clinical, radiological and pathological findings as well as the pulmonary functions have been described.

## 2. Case Report

A 55 year-old, nonsmoker male presented with a recurrent large left pneumothorax requiring a chest tube placement. He had a same side pneumothorax three and six years earlier, also requiring a chest tube placement. The patient also reported dyspnea on exertion with stairs and inclines, which had progressed over last three years along with a dry cough.

He had a remote exposure to quails while training hunting dogs. He was known to have hypertension and suffered with symptoms of gastroesophageal reflux disorder (GERD). His physical examination was unremarkable. A review of his pulmonary function revealed gradual decline in his FEV1, FVC and diffusion capacity over the past seven years (Table 1).

**Figure 1:**
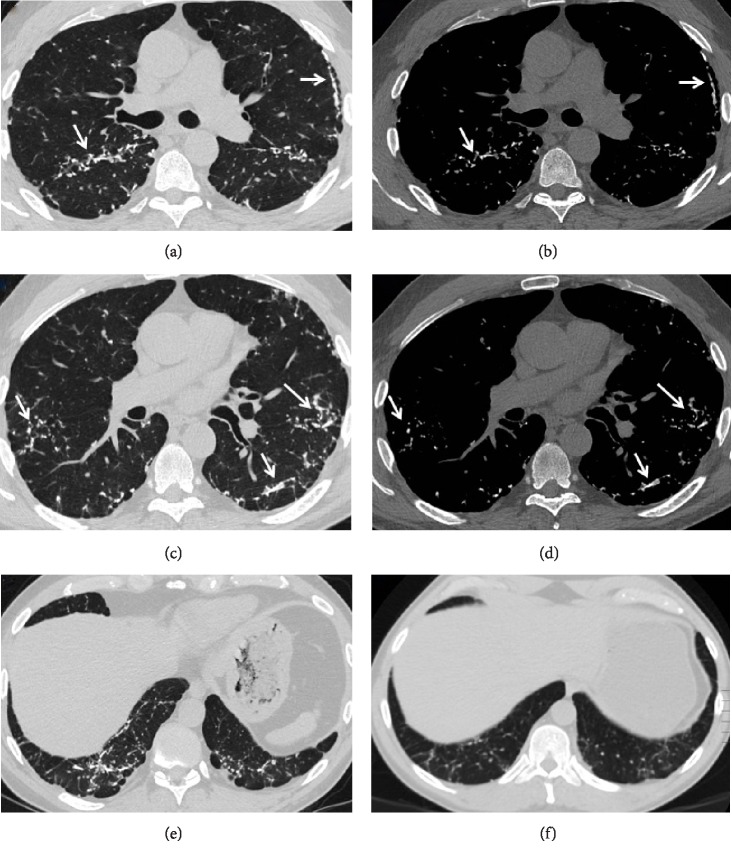
Axial chest CT images using lung (a), (c) and bone (b), (d) windows show bilateral branching dense nodular opacities (arrows) with mild associated reticulation. Some of the nodule are high in attenuation and almost iso-dense to ribs on bone windows. Axial images using lung widow (e), (f) at the level of lung bases were obtained 5 years apart and show evidence of progression.

**Figure 2:**
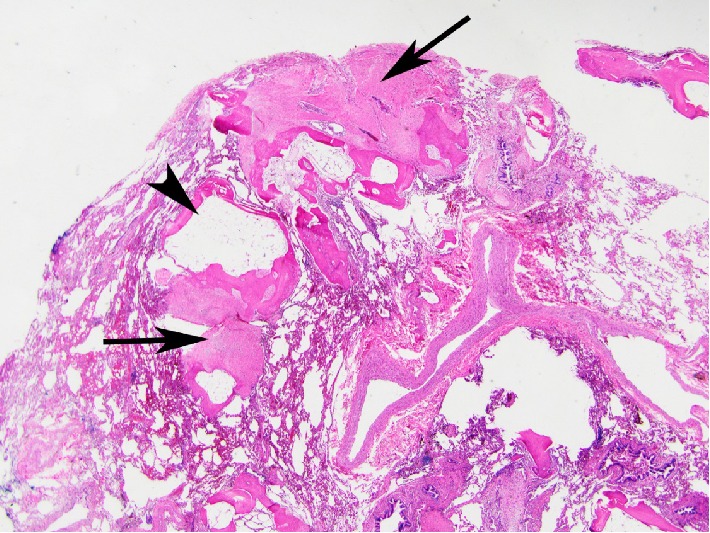
Large area of subpleural diffuse pulmonary ossification revealing lamellar bone with a more complicated pattern and areas of marrow elements including fat (arrowhead). Adjacent areas of cicatricial OP (arrows) are present (Hematoxylin and eosin; 12.5x).

**Figure 3:**
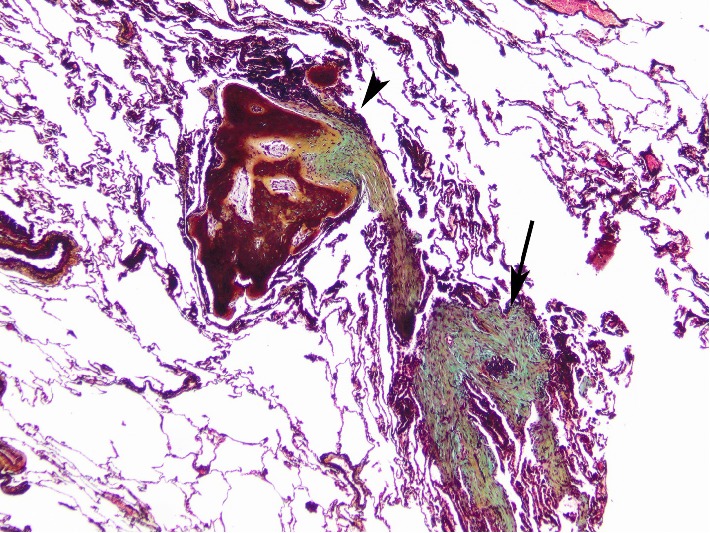
Areas of OP (arrow) with adjacent foci of cicatricial OP transitioning to DPO (arrowhead) (Movat stain; 20x).

**Figure 4:**
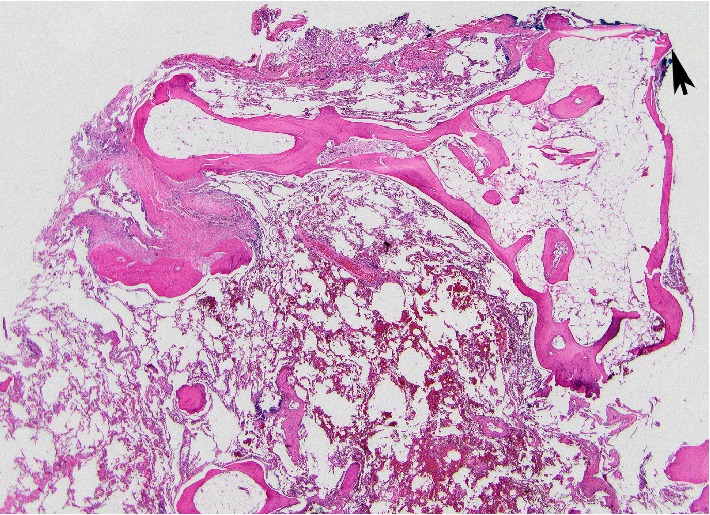
Focal areas of DPO in the subpleura with areas that appeared to extend through the pleura (arrow) (Hematoxylin and eosin; 40x).

Review of serial chest CT imaging revealed progressive bilateral sub-pleural and peribronchovascular branching dense opacities suggestive of DPO (Figure 1). He underwent VATS wedge resection of the left upper and lower lobes, which confirmed foci of dendriform ossification consisting of islands of ossifying fibrosis (Figure 2). In addition, there were scattered foci of OP, which, in some areas, had a more hyalinizing/cicatricial type morphology consistent with OPc. Many of the foci of DPO were associated with the OPc and showed transitions from conventional OP to OPc to the DPO (Figure 3). Dendriform pulmonary ossification foci were present in the subpleural region and occasionally adjacent to the visceral pleura (Figure 4). Given these imaging and pathological findings, the patient was diagnosed with DPO with cicatricial OP as the likely cause of his dyspnea, recurrent spontaneous pneumothoraces, and progressive restrictive impairment of lung function. Since there is no established medical treatment, his treatment plan was monitoring his symptoms and lung function and considering lung transplantation in case of progression.

## 3. Discussion

Organizing pneumonia is usually steroid-responsive and pathologically characterized by presence of loose fibromyxoid plugs within the lumens of the respiratory bronchioles and alveolar ducts [1, 2]. Cicatricial OP is distinguished from conventional OP by formation of irreversible dense fibrous bands and small nodules in a background of small or large foci of conventional OP [4]. In OPc, fibromyxoid plugs as seen in OP may be seen transitioning to more hyalinizing fibrous band/nodule of cicatricial OP and into foci of ossification on imaging as well as on pathology [4]. In our case, foci of DPO consisting of islands of ossifying fibrosis were present throughout both the left upper and lower lobes. In addition, there were scattered foci of hyalinizing/cicatricial type organizing pneumonia [4], representing a more chronic form of organizing pneumonia. Isolated focal plugs of hyalinized intra-alveolar tissue can be incidentally seen on in lobar resections for unrelated primary indications and should not confused with OPc. OPc tends to show bilateral and diffuse or patchy distribution on imaging [3, 4]. Fibrosing OP is a different from OPc, though it is not clearly determined what this pattern may represent. It may be form of disease where the fibrous tissues cause expansion of the alveolar septal interstitium such as in late organizing stages of diffuse alveolar damage or fibrotic forms of nonspecific interstitial pneumonia (NSIP) [3].

On CT, conventional OP presents as patchy bilateral peribronchial and subpleural consolidations [15–19], which may be migratory [20]. The classic “atoll” [20] or the “reverse halo” sign [18] is only seen in 20% cases manifesting as ground glass opacities with surrounding crescentic or ring-shaped consolidation [21] Perilobular opacities bordering the periphery of the secondary pulmonary nodules are observed [16, 19, 22] in more than half of OP cases. Manifestations of OPc include ground glass opacities, often associated with consolidations [15] and 1–10 mm nodular opacities with consolidation [16, 17]. Less common imaging features are subpleural or peribronchial irregular reticular opacities with areas of consolidation [15, 16, 19] and large nodules or mass-like consolidation [23]. Only 40% of the reported OPc cases show typical imaging appearance and other cases show variable nonspecific imaging findings including peribronchial and peripheral reticular opacities with or without evidence of pulmonary ossification [4]. Reticular opacities on CT imaging are seen in cicatricial OP cases where fibrous bands and nodules are the predominant feature on pathology with minor conventional OP. Presence of branching high densities on CT is suggestive of dendriform pulmonary ossification.

Pulmonary ossification is a metaplastic process where mature bone is present in the alveolar interstitium and/or alveolar spaces. Pulmonary ossification is classified into DPO and NPO. NPO is usually a localized process of lamellar bone and can occur in the setting of chronic congestion as seen in mitral valve stenosis. Unlike DPO, NPO usually does not contain bone marrow (fat or hematopoietic cells) [5]. On CT, NPO manifests as lower lobe predominant small, often highly attenuating, centrilobular nodules that may coalesce [24]. In DPO, more complicated tubular branching lamellar bone is seen, usually with marrow elements that can be seen in the alveolar space and expanding into alveolar septae [5]. DPO is commonly seen in the setting of chronic lung disease, UIP in particular [5, 6]. It has been described with cicatricial OP [4], and can be seen in isolation [7, 8].

DPO manifests on CT as small (several millimeters in diameter) nodules, which are seen predominantly in the peripheral interstitium (interlobular septa, subpleural and perifissural spaces). These nodules form contiguous and branching pattern resembling tree branches. In most instances these nodules are of high attenuation, reflecting the underlying ossification. The detection of small high-attenuation foci is improved using thin slices and maximum-intensity-projection images. DPO has tendency to affect lower lobes and posterior aspect of upper lobes. Association with recurrent aspiration and DPO has been suggested [7]. The presented patient had a history of GERD, which may be associated with recurrent episodes of aspiration. Pulmonary calcifications are commonly encountered on chest imaging and can be seen in several entities (Table 2) [8]. Distinction between pulmonary calcifications and ossification on imaging is not always possible. However, the distribution and the branching pattern are characteristic of DPO [7].

Spontaneous pneumothorax has been reported in several cases of DPO [9–14] including one case with cicatricial OP and DPO [4]. This case with cicatricial OP and DPO reported by Churg et al. [4] is likely the same case was reported by Tsai et al. [10]. In our case, foci of DPO were present in the subpleural areas, presumably causing recurrent pneumothoraces. It has been suggested that a subpleural sharp bony spicule may cause the pneumothorax by puncturing the visceral pleura [10, 13, 14]. None of the reported cases nor our case has other causes to explain the pneumothoraces, such as cystic or bullous lung disease, trauma, or bronchopleural fistula.

The case we present showed evidence of radiographic progression and worsening restrictive pattern on PFT over several years. The reported cases of cicatricial OP by Churg and his colleagues [4] have been suggested to be stable. However, almost half of those reported by Yousem [3] had persistent or progressive disease at a median of 110 months of follow-up. This may suggest that cicatricial OP is a spectrum and cases with minor conventional OP and predominant dense organization with fibrous bands and nodules may persist or even progress.

In summary, we present a case of cicatricial OP with DPO presenting with recurrent pneumothoraces and slow progressive pulmonary physiologic restrictive impairment. Cicatricial OP should be considered in the differential diagnosis of peribronchial or subpleural reticular opacities with DPO. Radiologists and clinicians alike should be aware of this newly described entity as distinct from other classical fibrosing processes, its potential association with DPO, and the presumed association between subpleural DPO and spontaneous pneumothorax.

## Figures and Tables

**Table 1:** Pulmonary function test over several years.

	FEV1		FVC		FEV1/FVC %	DLco	
	Value	Predicted %	Value	Predicted %		Value	Predicted %
2019	2.22	55%	3.01	57%	74	N/A	N/A
2017	2.62	69%	3.77	76%	70	20.74	68%
2015	2.96	76%	4.11	82%	72	24.76	80%
2014	3.15	81%	4.16	82%	76	24.76	80%
2013	3.41	87%	4.47	88%	76	23.61	76%
2012	3.39	85%	4.50	88%	75	32.39	103%

FVC: forced vital capacity, FEV1: forced expiratory volume in 1 second, DLCO: diffusing capacity of the lung for carbon monoxide (ml/min/mmHg), N/A: not available.

**Table 2:** Differential diagnosis of pulmonary calcifications and ossifications.^∗^

*Calcifications*	
Dystrophic: calcifications in diseased lung	(1) Infections: granulomatous infection such as histoplasmosis and tuberculosis and viral infections such as varicella
	(2) Granulomatous noninfectious disease: sarcoidosis
	(3) Occupational lung disease: silicosis, coal workers' pneumoconiosis
	(4) Metabolic lung diseases: amyloidosis, pulmonary alveolar microlithiasis
Metastatic: calcifications in normal lung	(1) Hypercalcemia in the setting of chronic renal failure, other causes of primary hyperparathyroidism, Paget's disease, parathyroid carcinoma or multiple myeloma
Calcified metastasis	(1) Metastases such as mucinous carcinoma, chondrosarcoma and synovial sarcoma

*Ossifications*	
NPO	In patients with chronic venous congestion such as long standing mitral stenosis
DPO	In patients with interstitial fibrosis
Bone forming neoplasms	Osteogenic sarcoma metastasis

^∗^Modified from reference [8]. NPO: nodular pulmonary ossification, DPO: dendriform pulmonary ossification.
